# Locoregional Treatment of Metastatic Pancreatic Cancer Utilizing Resection, Ablation and Embolization: A Systematic Review

**DOI:** 10.3390/cancers13071608

**Published:** 2021-03-31

**Authors:** Florentine E. F. Timmer, Bart Geboers, Sanne Nieuwenhuizen, Evelien A. C. Schouten, Madelon Dijkstra, Jan J. J. de Vries, M. Petrousjka van den Tol, Martijn R. Meijerink, Hester J. Scheffer

**Affiliations:** 1 Department of Radiology and Nuclear Medicine, Amsterdam University Medical Centers (Location VUmc), De Boelelaan 1117, 1081 HV Amsterdam, The Netherlands; b.geboers@amsterdamumc.nl (B.G.); s.nieuwenhuizen1@amsterdamumc.nl (S.N.); e.schouten@amsterdamumc.nl (E.A.C.S.); m.dijkstra3@amsterdamumc.nl (M.D.); j.devries1@amsterdamumc.nl (J.J.J.d.V.); mr.meijerink@amsterdamumc.nl (M.R.M.); hj.scheffer@amsterdamumc.nl (H.J.S.); 2 Department of Surgery, Amsterdam University Medical Centers (Location VUmc), De Boelelaan 1117, 1081 HV Amsterdam, The Netherlands; mp.vandentol@amsterdamumc.nl

**Keywords:** metastatic pancreatic cancer, oligo-metastases, locoregional treatment, resection, ablation, embolization

## Abstract

**Simple Summary:**

Metastatic pancreatic ductal adenocarcinoma (mPDAC) has a dismal prognosis. In selected patients with limited metastatic disease, locoregional therapy, in addition to systemic chemotherapy, may improve survival. This systematic review sought to examine current evidence on the value of additional locoregional treatment, including resection, ablation and embolization, in patients with hepatic or pulmonary mPDAC. The results, although liable to substantial bias, demonstrated superior survival from metastatic diagnosis or treatment in a subset of patients after radical-intent local primary and metastatic treatment (hepatic mPDAC 7.8–19 months; pulmonary mPDAC 22.8–47 months) compared to chemotherapy or best supportive care (hepatic mPDAC 4.3–7.6 months; pulmonary mPDAC 11.8 months). However, as a consequence of the bias, definitive conclusions regarding the seemingly beneficial effect of locoregional treatment cannot be endorsed. Randomized controlled trials with strictly selected oligometastatic PDAC patients are required to deduce final recommendations on this notion.

**Abstract:**

The prognosis of metastatic pancreatic ductal adenocarcinoma (mPDAC) remains universally poor, requiring new and innovative treatment approaches. In a subset of oligometastatic PDAC patients, locoregional therapy, in addition to systemic chemotherapy, may improve survival. The aim of this systematic review was to explore and evaluate the current evidence on locoregional treatments for mPDAC. A systematic literature search was conducted on locoregional techniques, including resection, ablation and embolization, for mPDAC with a focus on hepatic and pulmonary metastases. A total of 59 studies were identified, including 63,453 patients. Although subject to significant bias, radical-intent local therapy for both the primary and metastatic sites was associated with a superior median overall survival from metastatic diagnosis or treatment (hepatic mPDAC 7.8–19 months; pulmonary mPDAC 22.8–47 months) compared to control groups receiving chemotherapy or best supportive care (hepatic mPDAC 4.3–7.6 months; pulmonary mPDAC 11.8 months). To recruit patients that may benefit from these local treatments, selection appears essential. Most significant is the upfront possibility of local radical pancreatic and metastatic treatment. In addition, a patient’s response to neoadjuvant systemic chemotherapy, performance status, metastatic disease load and, to a lesser degree, histological differentiation grade and tumor marker CA19-9 serum levels, are powerful prognostic factors that help identify eligible subjects. Although the exact additive value of locoregional treatments for mPDAC patients cannot be distillated from the results, locoregional primary pancreatic and metastatic treatment seems beneficial for a highly selected group of oligometastatic PDAC patients. For definite recommendations, well-designed prospective randomized controlled trials with strict in- and exclusion criteria are needed to validate these results.

## 1. Introduction

Pancreatic ductal adenocarcinoma (PDAC) is one of the most lethal malignant neoplasms, with an overall 5-year survival rate of 6%. It is the fourth frequent cause of death from cancer in the USA and Europe, with deaths projected to increase in the forthcoming years [1]. The dismal prognosis can be attributed to its aggressive tumor biology, harboring immunosuppressive and chemoresistant traits. The treatment-insensitive tumor microenvironment is characterized by a desmoplastic stroma, acting as a physical barrier, and abundance of immunosuppressive molecules and immune cells [2,3]. Currently, the only potentially curative option is surgical resection of early-stage pancreatic tumors. Unfortunately, the majority (80%) of patients initially suffer from unspecific symptoms and present at an already advanced disease stage [4]. Frequent metastatic sites include liver, lungs, peritoneum and lymph nodes [5]. For metastatic PDAC (mPDAC, stage IV), the current standard of care is systemic palliative-intent chemotherapy. The median overall survival (mOS) with gemcitabine alone is 6.8 months, compared to chemotherapeutic combinations such as gemcitabine/nab-paclitaxel and FOLFIRINOX (folinic acid, fluorouracil, irinotecan and oxaliplatin) that achieve mOS outcomes of 8.5 and 11.1 months, respectively [6,7]. Within the scope of mPDAC, a subgroup of patients with limited disease burden seems to have a favorable outcome. Hellman and Weichselbaum were the first to propose the term ‘oligometastases’ in reference to an intermediary state between localized and disseminated metastatic disease. In this state, metastases are limited in number and restricted to a single or limited number of organs [8]. The authors postulated that this specific subset of patients could potentially benefit from additional locoregional treatments. Locoregional treatments for metastasized cancers, such as colorectal liver metastases [9,10] and metastatic pancreatic neuroendocrine tumors [11] have proven safe and effective, and are currently well established. However, in mPDAC, the local eradication of both the primary tumor and metastatic sites remains controversial due to its aggressive nature and consequential short survival. Nevertheless, over the last decade, improved surgical and interventional techniques have unlocked new potential for the locoregional treatment of mPDAC. These novel locoregional therapies include surgical, as well as minimally invasive image-guided therapies such as ablation and embolization. Ablative strategies utilize thermal or electrical energy, or highly focused radiation beams for the focal destruction of tumor tissue, and include, but are not limited to, radiofrequency ablation (RFA), irreversible electroporation (IRE), stereotactic body radiotherapy (SBRT), and high-intensity focused ultrasound (HIFU). Furthermore, embolization techniques, including selective internal radiation therapy (SIRT), also known as transarterial radioembolization (TARE) and transarterial chemoembolization (TACE), aim to locally deliver synthetic embolic agents, simultaneously blocking the tumor’s blood supply. These minimally invasive and image-guided interventional approaches broadened the treatment spectrum for many solid tumor types [12,13,14,15]. An interesting next step would be to explore whether locoregional treatment approaches prove to be beneficial for a subset of patients with oligometastatic PDAC.

This systematic review focuses on locoregional treatments, including resection, ablation and embolization, for mPDAC with a focus on hepatic and pulmonary metastases. The aim is to explore and evaluate current evidence on the use of these locoregional treatments in mPDAC. Study details and outcome parameters will be highlighted and compared. The investigated parameters encompass patient and disease characteristics, local and systemic treatments, prognostic factors, morbidity and mortality, and overall survival. Factors to consider when setting up a randomized controlled trial (RCT) on the topic of this systematic review will be discussed. Finally, future directions regarding multimodality treatments integrating locoregional treatment and immunotherapy will be presented.

## 2. Materials and Methods

This review was written according to the Preferred Reporting Items for Systematic Reviews and Meta-Analyses guidelines for reporting systematic reviews [16]. A comprehensive systematic search of the PubMed database was conducted that included studies from 2005 until September 2020. The search terms included (“pancreatic cancer” OR “pancreatic adenocarcinoma” OR “pancreas adenocarcinoma” OR “pancreatic ductal adenocarcinoma”) AND (metastatic OR metastases OR oligometastatic) AND (surgery OR resection OR metastasectomy OR ablation OR radiofrequency ablation OR irreversible electroporation OR stereotactic body radiotherapy OR high intensity focused ultrasound OR cryoablation OR microwave ablation OR embolization OR transarterial radioembolization OR selective internal radiation therapy OR SIRT OR TARE OR transarterial chemoembolization OR TACE). The ‘similar articles’ or ‘cited by’ functions in PubMed were used to further broaden the search. Additionally, recent review articles on this topic were searched for additional articles. Exclusion criteria included review articles, conference abstracts, articles not in English, case reports with fewer than 5 relevant patients, unspecific focus on a subset of liver-directed therapy (LDT), downstaged mPDAC and other tumors located in the pancreas (neuroendocrine, periampullary or metastases of other primary tumors).

### Definitions

The disease-free interval (DFI) is defined as the time from local pancreatic treatment until unequivocal local and/or distant disease progression. Unless stated otherwise, the timing of chemotherapy administration (i.e., neoadjuvant or adjuvant) is described from the perspective of metastatic treatment, not primary treatment. Thus, in case of metachronous disease, neoadjuvant therapies refer to those administered after primary pancreatic treatment and prior to metastatic treatment. Adjuvant refers to those treatments given after local metastatic interventions. Local tumor control in the liver is defined as the percentage of stable disease (SD), partial response (PR) and complete response (CR) based on the RECIST criteria [17]. Complications are reported according to the Common Terminology Criteria of Adverse Events (CTCAE), in which grade 3 or higher appertains to a serious adverse event (SAE) [18].

## 3. Results

**Figure 1:**
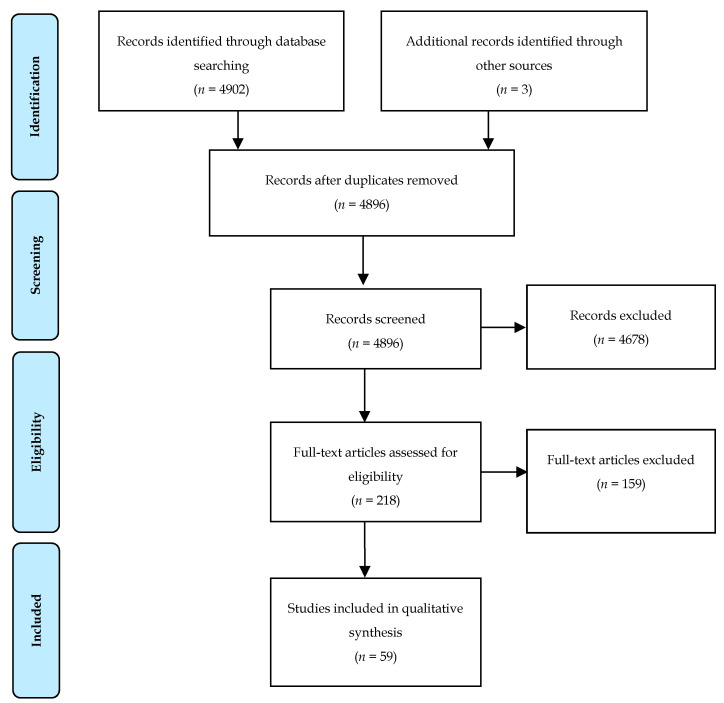
Flow diagram of the systematic search according to PRISMA [16].

The search identified 4902 articles (Figure 1). After removal of articles that did not meet the inclusion criteria, a total of 59 articles were selected on the topic of locoregional treatment in the context of mPDAC. Of these, 35 articles discuss the use of resection, 10 focused on ablative techniques and 14 present details on the use of embolization. No articles specifically focused on cryoablation or microwave ablation. In total, 63,453 patients were included: 61,035 patients from the Surveillance, Epidemiology and End Results (SEER) population database and 2418 patients from non-SEER database articles.

### 3.1. Resection

#### 3.1.1. Primary Pancreatic Tumor Resection

Five large database studies focused on the effects of surgical removal of the primary pancreatic tumor in case of metastatic disease (Table 1) [19,20,21,22,23]. They included a total of 61,035 patients from the SEER database. Of these patients, 1217 underwent resection of their primary pancreatic tumor with the remaining 60,818 non-primary resected patients serving as controls. Overall survival (OS) in the resection group, regardless of metastatic location, ranged between 4.7 and 14 months, whereas the OS of the non-resected patients varied between 2 and 9.1 months. However, baseline characteristics including age, primary tumor size and location, lymph node status and prior chemoradiation mostly favored resected patients, inherently introducing selection bias and likely affecting the survival outcomes. Wang et al. [22] were the only group to incorporate propensity score matching (PSM 1:1) in order to match the resected (*n* = 365) and non-resected (*n* = 365) patient cohorts based on the baseline characteristics, significantly substantiating their findings. They found the median OS (mOS) to be significantly (*p* < 0.05) longer in resected (11.6 months) compared to non-resected (9 months) patients. Although patient numbers are large in these five SEER database articles, a limitation is the presumably present patient record duplication, since similar cohorts from overlapping years were targeted. In addition, none of the studies reported details on procedure-specific morbidity and mortality rates, which is a crucial indicator for practical implementation. McKenzie et al. [19] demonstrated the lowest survival outcomes in resected patients (6.3 months). Importantly, a large subgroup of patients did not receive additional chemotherapy. They concluded that resection improves the median survival solely in patients receiving chemotherapy in addition to resection (9 vs. 4.7 months, *p* < 0.001), denoting the importance of systemic chemotherapy in the treatment of metastatic pancreatic cancer. Prognostic factors (Table 2 that positively influenced survival included those based on patient characteristics, disease characteristics (i.e., lower histological grade, longer disease-free interval (DFI), single-organ metastatic disease) and receiving chemotherapy, primary resection or local metastatic treatment.

#### 3.1.2. Hepatic Metastasectomy

The majority of studies on liver-directed therapy (LDT) in mPDAC concern the surgical removal of liver metastases in addition to primary resection (Table 3) [24,25,26,27,28,29,30,31,49,50,51,52,53,54,55,56,57,58]. A total of 18 articles were identified on this topic including 938 patients with mPDAC spread to the liver, of whom 528 received primary pancreatic resection in combination with hepatic metastasectomy. The remaining 410 patients served as controls and had received sole local primary or metastatic treatment (*n* = 77), undergone surgical exploration (with or without palliative bypass) (*n* = 190) or were treated with chemotherapy (*n* = 143). A trend of increased survival from metastatic diagnosis or treatment was observed in patients treated with primary resection and hepatic metastasectomy (mOS 7.8–14.5 months), compared to patients receiving sole primary resection (mOS 9.2 months), sole local metastatic treatment (7.5 months) or exploration/chemotherapy (4.3–7.6 months). Tachezy et al. [24] reported on the largest patient cohort (*n* = 138) with matched controls based on baseline characteristics, significantly substantiating their findings. Half the cohort (*n* = 69) received pancreatic tumor resection and synchronous hepatic metastasectomy, the other half (*n* = 69) received liver metastasectomy without pancreatic resection and served as matched controls. OS for the resection group compared to the controls was significantly increased at 14.5 vs. 7.5 months, respectively (*p* < 0.001). These results indicate that local surgical treatment of metastases only benefits patients whose primary pancreatic tumor is resected. OS outcomes after metachronous hepatic metastasectomy (and prior pancreatic resection) ranged between 11.4 and 36.8 months (from metastatic diagnosis or treatment) [30,52,58]. This major discrepancy may be a consequence of low patient numbers in combination with variable inclusion criteria (i.e., resection margin). Bahra et al. [54] specifically stratified survival outcomes on the basis of the primary resection status. The mOS of mPDAC with R0, R1 and R2 margins were 14.4, 7.3 and 6.1 months, respectively, demonstrating the substantial influence of the pancreatic resection margin status on survival. Another factor that varied greatly between studies that likely influenced survival is the chemotherapeutic regimen. The majority reported less than 20% of patients receiving neoadjuvant chemotherapy [24,25,27,31,55], whilst some used it as an inclusion criterion, thus reaching 100% [28,56,57]. Adjuvant chemotherapeutic regimens also varied substantially, between 9% and 100%. This discrepancy significantly reduces the comparative power between studies. Grade 3+ morbidity varied between 3% and 20% for synchronous resections, and between 12% and 25% for metachronous liver procedures. Most common and relevant complications included pancreatic fistulas, hemorrhages, infections, delayed gastric emptying and intra-abdominal abscesses. Eight studies reported peri-operative mortality rates, the highest rate being 9.1% [50]. Positive prognostic factors (Table 2) included primary and metastatic disease characteristics such as a lower histological grade, smaller (primary and metastatic) tumors and lower tumor marker CA19-9 serum levels, as well as treatment-related factors. The multitude of pancreatic-related predictors of survival underline its importance as a determining factor in a patient’s suitability for locoregional treatment in a metastatic setting.

#### 3.1.3. Pulmonary Metastasectomy

Within the context of mPDAC, we identified 12 retrospective studies that describe pulmonary metastasectomy (Table 4) [32,33,34,35,36,59,60,61,62,63,64,65]. They included a total of 318 PDAC patients with single-organ metastatic disease to the lungs (14 synchronous, 292 metachronous, 12 unknown), of whom 143 received primary pancreatic resection later followed by pulmonary metastasectomy. The remaining 175 served as controls and were given sole local pancreatic or pulmonary treatment, chemo(radio)therapy (CRT) or best supportive care (BSC). Primary resection followed by pulmonary metastasectomy (22.8–47 months) demonstrated significantly improved mOS from metastatic diagnosis or treatment, compared to exclusive pancreatic resection (8.1–20.2 months), solitary metastatic treatment (10.7 months) or CRT/BSC (11.8 months). Yasukawa et al. [63] reported an exceptionally high mOS (121 months from primary diagnosis) by including a highly selected group of patients (*n* = 12) with long DFI after initial pancreatic resection, isolated stable disease over time and a favorable response to systemic therapy. Arnaoutakis et al. [59] were the only group to include semi-matched controls based on age and disease burden at the time of recurrence. They demonstrated superior survival from primary diagnosis for patients with lung-only metastatic disease receiving primary and pulmonary resection compared to controls receiving sole primary resection (mOS 51 vs. 23 months; *p* = 0.04). However, it must be noted that the two treatment groups did significantly (*p* < 0.001) differ on the basis of median DFI. Synchronous disease outcomes were reported by Kruger et al. [35], who achieved a mOS of 22.8 months from primary diagnosis for patients receiving both primary and pulmonary resection. In addition, they reported mOS outcomes (10.7 months) of metachronous mPDAC patients with a locally advanced pancreatic cancer (LAPC), who received primary CRT later followed by pulmonary metastasectomy. None of the articles reported grade 3+ complications or procedure-related mortalities. All but one study reported neoadjuvant (i.e., prior to pulmonary metastasectomy) chemotherapy rates of at least 70%. Adjuvant chemotherapeutic regimens were less well documented and had greater variability, ranging between 29% and 100%. Amongst the studies, favorable prognostic factors (Table 2) included a longer DFI, fewer lesions, well/moderate differentiation of the tumor, and lower tumor marker CA19-9 levels prior to primary resection or at the time of recurrence. Surprisingly, in contrast to the articles on hepatic metastasectomy in mPDAC, none of the articles reported primary resection margin status or tumor stage at time of presentation as significant prognostic factors. Overall, pancreatic cancer patients with (synchronous or metachronous) sole metastatic spread to the lungs define a specific subgroup with opportune prognosis. They demonstrate significant survival benefit over other metastatic sites, reflecting the apparent favorable tumor biology [66].

### 3.2. Ablation

#### 3.2.1. Radiofrequency Ablation (RFA)

Three articles were published that incorporated RFA in the treatment of mPDAC, all of which employed the technique for hepatic metastatic treatment (Table 4) [38,39,40]. They included a total of 262 patients with liver mPDAC (108 synchronous, 154 metachronous disease), of whom 196 had their hepatic lesions treated with RFA and 66 received palliative chemotherapy, serving as controls [38,39,40]. Slightly improved survival (from metastatic diagnosis or treatment) was observed in mPDAC patients receiving primary resection and liver-directed RFA (12–14 months) and patients receiving liver-directed RFA only (11.4 months), compared to a matched chemotherapy control group with metachronous disease (9.1 months). Variability regarding patient characteristics among the studies such as disease timing (i.e., synchronous or metachronous) and chemotherapeutic regimens likely impacted survival. For example, the percentage of patients receiving (neo)adjuvant chemotherapy ranged from 62% to 100%, creating an influential confounder. Grade 3+ complication rates amongst studies varied between 10% and 13%, none reporting peri-procedural mortality. Those of clinical relevance included pleural effusions, liver abscesses and hemorrhages. All three articles reported prognostic factors (Table 2), those offering a better survival outcome including fewer [40] and smaller liver lesions [38,39,40], well/moderate differentiation of the tumor, longer DFI [38] and a lower primary tumor stage [38].

#### 3.2.2. Irreversible Electroporation (IRE)

To date, only one article has been published on IRE for mPDAC (Table 5) [67]. Hong et al. included 7 patients with synchronous metastatic lesions and reported a median OS of 16 months from initial local treatment. Open and percutaneous IRE were employed for either primary pancreatic or metastatic treatment. The metastatic lesions were located in the liver (*n* = 4), omentum (*n* = 3) and peritoneum (*n* = 3). Complications were not reported, and peri-operative mortality was 0%. All patients received neoadjuvant chemotherapy, and 57% continued adjuvant therapy post IRE.

#### 3.2.3. Stereotactic Body Radiotherapy (SBRT)

Five articles were published on the use of SBRT for mPDAC (Table 5). This ablative technique was utilized for both primary pancreatic [41,68,69,70] as well as metastatic treatment [42,70]. The articles included 106 patients with mPDAC (53 synchronous, 39 metachronous disease, 14 unknown). The mOS from SBRT treatment varied between 8.5 and 23 months, with none of the articles mentioning a control group. The lowest survival outcomes were reported by Su et al. [69] (mOS 8.5 months). They solely treated the primary tumor with SBRT without locally treating the metastases. In addition, their (neo)adjuvant chemotherapy rates were low (8%). The combination of these factors likely explains the low survival outcomes. The highest survival outcome (mOS 23 months) was reported by Scorsetti et al. [42], which was probably caused by their inclusion of patients with lung-only metachronous disease. Due to the heterogeneity among the few articles in terms of metastatic sites, pancreatic tumor resection, inclusion of LAPC and the dual use of SBRT treatment (pancreatic vs. metastatic), survival outcomes cannot be directly compared. For example, Gkika et al. [70] and Scorsetti et al. [42] included patients with pulmonary (*n* = 12) and hepatic ± lymph node (*n* = 42) metastases, whereas the other three articles did not specify the metastatic sites. The variability in chemo-(8–100%) and radiotherapy (20–75 Gy) regimens add to the list of features limiting comparative power. Highest reported clinically relevant (grade 3+) acute (<90 days) or late (>90 days) toxicity was 6% [70]. Among these studies, the most relevant acute toxicities included mechanical ileus, gastro-intestinal bleeding, and relevant late toxicities included hemorrhage, gastric outlet obstruction and gastroduodenal ulcer. Positive prognostic factors (Table 2 in terms of OS reported amid these studies included smaller lesion sizes and longer DFI.

#### 3.2.4. High-Intensity Focused Ultrasound (HIFU)

Similar to IRE, HIFU has not been widely analyzed as a local ablative tool in the context of mPDAC (Table 5). One article was published by Li et al. [37], in which a total of 120 gemcitabine-refractory patients were included with metastatic lesions in the liver (*n* = 76), lung (*n* = 59) and/or peritoneal lymph nodes (*n* = 57). They reported a significant (*p* < 0.001) survival benefit for patients receiving HIFU of their metastases in addition to systemic chemotherapy (*n* = 61, mOS 10.3 months) compared to chemotherapy treatment alone (*n* = 59, mOS 6.6 months). However, survival was not stratified based on metastatic location. In both groups, around half the patients had undergone prior primary pancreatic resection. They reported 68 minor complications in 60 patients, none experiencing grade 3+ morbidity. Furthermore, there was no peri-procedural mortality. Favorable prognostic factors (Table 2) included age < 65, 0–1 performance score, previous pancreatic resection, well/moderate differentiation of the tumor, absence of liver metastases, and fewer lesions.

### 3.3. Embolization

#### 3.3.1. Selective Internal Radiation Therapy (SIRT)/Transarterial Radioembolization (TARE)

Eight articles were identified that utilized SIRT, also known as TARE, for hepatic metastases in mPDAC (Table 6) [44,45,46,47,71,72,73,74]. A total of 302 patients with mPDAC (136 with liver only disease, 166 with liver + extrahepatic disease (EHD)) were included, of whom 59 were treated with pancreatic resection or SBRT and SIRT of the liver, 93 received SIRT of the liver only, 120 were used as matched controls receiving chemotherapy only, and 30 others (unknown primary treatment or different local metastatic treatment). The mOS from SIRT in mPDAC patients ranged between 5.5 and 13.6 months, compared to a mOS of 6.3 months from primary diagnosis in the matched controls who received exclusive systemic chemotherapy. However, most of these (lower) survival outcomes after SIRT include non-stratified data of patients with and without primary pancreatic resection and with and without EHD, hence limiting their power. In this regard, Gibbs et al. [72] demonstrated meaningful results by stratifying their results based on primary surgical treatment. They found that patients who received both primary resection and SIRT of the liver had significantly improved OS compared to those receiving SIRT only (mOS 13.6 vs. 4.2 months, respectively; *p* = 0.015). Moreover, Ouyang et al. [44] specifically reported the survival outcomes of patients (*n* = 64) receiving SIRT (or other LDT) without primary resection, achieving a mOS of 8.7 months, which is also considerably lower than the 13.6 months reported by Gibbs et al. These results indicate that local treatment of the liver only improves survival when combined with primary pancreatic resection. All studies reported neoadjuvant chemotherapy rates of at least 80% (up to 100%). Adjuvant chemotherapeutic regimens were reported in two articles, their rates spanning 30–47%. Three groups incorporated concomitant regimens (94–100%) into their treatment protocol. The reported complications varied substantially in type and rates, with some reporting acute and late toxicities, other clinical adverse events and biochemical toxicities. The clinically relevant (i.e., grade 3+) complication rates ranged between 0% and 64%, with SIRT-related mortality up to 16%. Articles that integrated concomitant chemotherapy noted, on average, higher relevant complication rates. Most relevant favorable prognostic factors (Table 2) included smaller primary tumor size, previous pancreatic surgery, LDT over chemotherapy alone, lower CA19-9 prior to SIRT and solitary lesion (vs. multiple).

#### 3.3.2. Transarterial Chemoembolization (TACE)

Seven articles reported findings on LDT utilizing TACE in metastatic pancreatic cancer (Table 6) [43,44,48,75,76,77,78]. A total of 549 of mPDAC patients were included (416 with liver-only, 109 with liver and EHD, and 24 liver with or without EHD). These patients received primary resection or RFA or TACE in combination with TACE of the liver (*n* = 273), TACE of the liver only (*n* = 31), TACE of the liver with unknown local primary treatment (*n* = 24), other LDT (*n* = 33), or chemotherapy only (*n* = 188, serving as matched controls). The mOS from (the first) TACE in mPDAC patients who had their primary tumor resected previously varied between 7.5 and 19 months. For comparison, the only article including a control group with similar baseline characteristics who received systemic chemotherapy only achieved a mOS of 6.3 months from primary diagnosis, indicating a survival benefit for patients receiving pancreatic resection and TACE [44]. Interestingly, Vogl et al. [77] demonstrated the highest mOS (19 months from the first TACE) whilst including patients who progressed under systemic chemotherapy with the majority bearing multi-metastatic (*n* ≥ 5 lesions) disease. Their survival outcomes can possibly (in part) be explained by the selection of patients without EHD and the utilization of an efficacious triple chemotherapeutic drug combination, as is reflected by their substantial local liver control (93%). Neoadjuvant chemotherapy rates (13–100%) varied greatly between studies, likely creating a confounder, as was illustrated in the article by Kim et al. [75]. They reported the lowest survival outcomes, which may be explained by their low systemic chemotherapy rates (13%) and inclusion criteria. They included patients deemed unsuitable for RFA due to tumor size, number or location, inherently selecting less favorable patients as was portrayed by their relatively low local liver control (67%). Clinical grade 3+ morbidity rates varied substantially, ranging between 0 and 70%, with one possible TACE-related death. Most relevant complications included liver abscesses, vomiting/nausea and pain. Independent prognostic factors (Table 2) that positively correlated with survival included male gender and absence of EHD.

## 4. Discussion

The prognosis of metastatic PDAC (mPDAC) remains universally poor. Hence, new treatments are sought that further prolong survival without further compromising quality of life in these patients. An oligometastatic state in patients with PDAC, in which metastatic disease is limited in number and restricted to single or limited number of organs, possibly allows for additional locoregional treatment.

Patients with oligometastatic PDAC (primarily) spread to the liver or lungs who received both radical-intent local primary and local metastatic treatment (liver 7.8–19 months; lungs 22.8–47 months) had superior survival outcomes from metastatic diagnosis or treatment compared to those receiving chemo(radio)therapy (CRT) or best supportive care (BSC) (liver 4.3–7.6 months; lungs 11.8 months) (Table 7) [26,27,50,52,57,66,78]. For hepatic mPDAC, neither exclusive local primary (9.1–9.2 months) nor sole local metastatic (7.5 months) treatment seems beneficial when compared to the CRT or BSC survival outcomes. This also holds true for solitary local metastatic treatment (10.7 months) in pulmonary mPDAC. Sole primary resection (8.1–20.2 months) in pulmonary mPDAC, although seemingly beneficial, only concerned metachronous cases, implying the resection took place in a non-metastasized setting. Thus, these results do not provide information regarding sole primary resection in case of pulmonary mPDAC. It is evident that a potential survival benefit would exclusively pertain to patients undergoing both radical-intent local primary and metastatic treatment. The majority of studies included in this systematic review endorse this notion. However, it is important to note that these results are strongly subject to selection bias and, hence, likely portray a skewed scenario. Limitations of these studies include of the frequent retrospective and non-randomized design, significant inter- and intra-heterogeneous patient populations and various inclusion criteria, inevitably leading to bias and confounders. Moreover, patient cohorts were often small and had no equivalent control groups, substantially limiting that study’s power. When comparing the various locoregional treatments, no apparent distinction can be made between resection, ablation and embolization in terms of overall survival due to the great variability between and restricted number of studies on ablation or embolization techniques. Serious adverse events (grade 3+) and (procedure related) mortality varied greatly between hepatic metastasectomy (3–25%; 0–9%), pulmonary metastasectomy (0%; 0%), hepatic ablation (0–13%; 0%) and hepatic embolization (0–64%; 0–16%). Especially pulmonary resection seemed uncomplicated based on these results. Local treatment in case of hepatic disease had a broad range of complications and mortality, depending on the utilized technique. Ablation seems preferential for treating hepatic disease, whereas embolization resulted in a large number of major complications and death. However, embolization is typically employed in case of abundant liver disease, naturally provoking a selection bias by including patients with more advanced stage metastatic disease. The potential individual roles of these techniques in the treatment of mPDAC will need to be determined in future clinical trials.

Remarkably, patients harboring metastatic disease confined to the lungs performed significantly better in terms of survival compared to any other metastatic site. The link between location of metastatic spread and survival in pancreatic cancer is in line with previous literature [66,79,80]. The correlation can be explained by favorable genetic mutations (i.e., less aggressive tumor biology) in micrometastases able to home the lungs and by the fact that pulmonary metastases often present later (metachronous) in the disease progression of PDAC compared to other metastatic sites [81,82]. For any metastatic site, synchronous detection of metastatic lesions indicates a more advanced disease state compared to metachronous disease. The survival benefit of metachronous disease was confirmed by the plethora of studies who reported the DFI as a significant prognostic factor [23,32,34,36,38,42].

**Figure 2:**
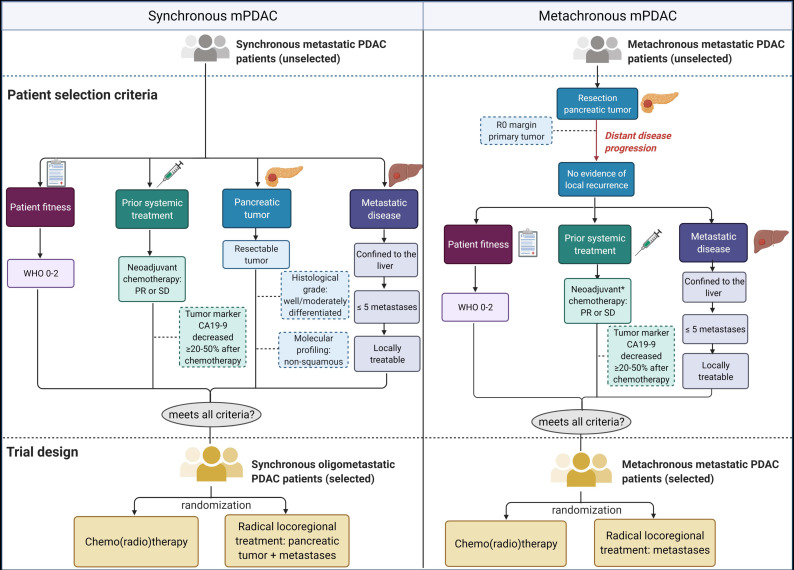
Randomized controlled trial (RCT) design. Two potential setups of an RCT to determine the possible life-prolonging value of locoregional treatment in synchronous (left) or metachronous (right) metastatic PDAC (mPDAC). The RCTs adhere to four main selection pillars: patient fitness, prior systemic treatment, pancreatic tumor and metastatic disease. Selection criteria include WHO performance status 0–2, partial response (PR) or stable disease (SD) after neoadjuvant chemotherapy, having a resectable primary tumor (synchronous mPDAC), metastatic disease confined to the liver and ≤5 metastases that are locally treatable. For metachronous mPDAC, the pancreatic tumor has to be resected previously, without evidence of local recurrence. In addition to the main criteria, several supportive selection criteria are portrayed (dotted outline), which may or may not be used, including a decrease of ≥20–50% in tumor marker CA19-9 serum levels after chemotherapy, lower histological grade (well/moderately differentiated), non-squamous transcriptomic subtype and, in case of metachronous mPDAC, an R0 resection margin of the primary tumor. If mPDAC patients meet all these requirements, they can be randomized into either a radical locoregional treatment group or a control group receiving chemo(radio)therapy. * After primary resection but prior to metastatic treatment.

It should be emphasized that the potential survival benefit of additional locoregional treatments solely applies to a highly selected group of mPDAC patients, thus making proper patient selection a crucial element. Based on the presented results as well as previous discussions [83,84], this is particularly true for patients with hepatic mPDAC, since a potential survival benefit is uncertain, but risk of procedure-related morbidity and mortality is high. Although patients with pulmonary mPDAC should also be selected carefully, local treatment seems more justified, with a more apparent survival benefit and fewer complications. Prognostic factors (Table 2) presented in the analyzed studies give an insight into which patient and tumor characteristics can be utilized for this selection. Based on this information, we have constructed two possible setups of RCTs for patients with synchronous or metachronous mPDAC who, upon meeting all selection criteria, can be randomized into either the radical locoregional treatment group or control group (CRT) (Figure 2). The included criteria are discussed below.

Although the oligometastatic state does not exclude multiple affected organs, multi-organ disease does significantly decrease survival [23,38,45,48]. Hence, a preference must be given to patients with single-organ disease. In reference to our previous statement, we believe there is a great need to explore the potential benefits of additional locoregional therapy in hepatic mPDAC patients and, hence, our RCTs focus on this subgroup. Still, a similar concept could be applied for a trial including pulmonary mPDAC patients. As for the metastatic disease load, the number of lesions was a commonly noted prognostic factor [28,34,35,37,39,40,47]. We propose a cut-off of 5 lesions, with the explicit criterium that all lesions can be radically treated with locoregional therapy. Based on the superior survival outcomes of patients whose pancreatic tumor was resected, a selection criterium for inclusion should be a resectable primary tumor in case of synchronous disease or a previously resected primary tumor in case of metachronous disease. Systemic chemotherapy remains an important tool in multimodality treatment of mPDAC. It can effectively be used for cytoreduction, with the potential of downstaging, to increase safety and efficacy of subsequent locoregional therapies [85]. Furthermore, the chemotherapy-induced tumor response could be used as an initial selection criterium, excluding those with progressive disease during treatment [25,28,56,63]. In PDAC, the most significant predictor of long-term survival is an R0 resection of the primary tumor since this remains the only possible curative option. Primary resection (margin) status was a frequently mentioned significant prognostic factor [20,21,22,23,24,28,31,37,46,54], confirming its substantial influence on survival and highlights its advantage as patient selection criterium [26,52,86]. However, this is specifically the case for metachronous disease, as for synchronous disease intra-operative efforts must be made to attain this information. Other disease-related features reported as positive predictors of survival include a lower histological grade (well/moderately differentiated) [25,32,37,38,40] and lower (pre- and/or post-treatment) tumor marker CA19-9 serum levels [28,33,34,45,48]. These markers give an indication of disease progression and tumor aggressiveness, and thus impact prognosis. Tumor marker CA19-9 serum levels that have decreased ≥20–50% after chemotherapy are associated with a positive tumor response and improved survival [87]. These two markers may potentially be used for selection and could therefore be considered supportive selection criteria. In addition to these currently known and commonly utilized clinical biomarkers, novel and more specific biomarkers are required to further advance personalized treatment strategies for mPDAC. One such biomarker is the molecular subtype of cancer. Molecular subtyping stratifies PDAC based on molecular data and commonly utilizes transcriptomics, which reflects substantial epigenetic changes. PDAC transcriptomic subtypes harbor a unique mutational profile that is linked to survival outcomes. Herein, squamous subtypes are associated with poor prognosis, and could thus serve as patient exclusion criterium [88].

Besides the careful and precise selection of PDAC patients with oligometastatic disease suitable for integrated systemic and locoregional therapy, prospective multimodal strategies should focus on incorporating novel treatment strategies. Several ablative therapies have shown to induce a systemic anti-tumor immune response in pancreatic cancer [89]. Combinatory strategies that integrate immunotherapy with ablative techniques have shown promising pre-clinical and clinical results for PDAC [90,91,92,93,94,95]. Narayanan et al. [92] reported on a pre-clinical study involving immunocompetent mice that received a combination of IRE, a checkpoint inhibitor and Toll-like receptor agonist. Compared to IRE alone, this combination resulted in an improved treatment response and elimination of an untreated concomitant metastasis. These encouraging results will be translated into a clinical study: the PANFIRE-III trial (NCT04612530).

## 5. Conclusions

Multimodality treatment for oligometastatic PDAC, incorporating systemic chemotherapy and radical locoregional therapy of both the primary and metastatic lesions, appears to be beneficial in a highly selected subgroup of patients with favorable disease characteristics. However, given the limitations of the presented studies, these results are liable to substantial bias and thus demonstrate trend indications rather than conclusions on the effects of additional locoregional therapy. Hence, we conclude that, at this moment in time, locoregional treatment for mPDAC should not be provided outside the context of an experimental trial. The focus should be on instituting large prospective randomized controlled trials (RCTs), potentially using the aforementioned design with suggestions on patient selection criteria. Such trials may confirm the results presented in this systematic review and identify the value of the individual locoregional therapies within the treatment landscape of mPDAC.

## Figures and Tables

**Table 1:** Primary tumor resection in mPDAC. NS = not specified; R = retrospective; P = primary resection; NR = no resection; M = metastatic resection (unknown whether this was in combination with primary resection); PM = primary + metastatic resection; PSM = propensity score matching.

Authors	Year	Design	Nr. of Patients (mPDAC)	Study Details	Synchronous/Metachronous	Morbidity, Grade 3+	Mortality	Chemotherapy	Median Overall Survival (Months)
McKenzie [19]	2010	R	4649	92 P 4557 NR	92/0	NS	P: 0–17.5 NR: 5.8–34.1%	P: 36% NR: 38.4%	From primary res: P: 6.3 NR: 4.9
Tao [20]	2017	R	28,918	467 P 28,451 NR	NS	NS	NS	P: 53% NR: 45%	From primary diagnosis: P: 7 NR: 2
Oweira [21]	2017	R	13,233	160 P 504 M 14,812 NR	NS	NS	NS	NS	NS
Wang [22]	2019	R	2694	365 P 2329 NR	NS	NS	NS	PSM 1: P: 69% NR: 66%	From primary res (PSM 1): P: 11.6 NR: 9
Liu [23]	2020	R	11,541	133 P 24 PM 6999 NR	NS	NS	NS	NS	From primary diagnosis: P: 10–14 NR: 4–6

**Table 2:** An overview of positive prognostic factors per article category (primary resection, hepatic resection, pulmonary resection, ablation and embolization).

Treatment	Positive Prognostic Factors— Patient Characteristics	Positive Prognostic Factors— Disease Characteristics (Primary Tumor)	Positive Prognostic Factors— Disease Characteristics (Metastases)	Positive Prognostic Factors—Treatment(s)
Primary resection	Younger age [20,21] Caucasian ethnicity [20,21] Married [20,21] Female [20,21] Later era of diagnosis [22]	Tumor in pancreatic head [20] Well/moderate differentiation of tumor (histological grade) [20,22,23]	Longer DFI [23] Single-organ metastatic disease [23]	Chemotherapy (pre- and/or post local treatment) [20,22] Primary tumor resection [20,21,22,23] Local metastatic treatment [21]
Hepatic resection		Lower tumor stage [24,25] Tumor in pancreatic body/tail [26] Smaller primary tumor size [27] Well/moderate differentiation of tumor (histological grade) [25] Lower tumor marker CA19-9 levels pre- or post-treatment(s) [25,28,29]	Fewer and/or smaller liver lesions [28] Metachronous (instead of synchronous) [30] Absence of lymph node status [25,31]	Chemotherapy (pre- and/or post local treatment) [25,28,29] Primary tumor resection [28,29] Better primary resection status [24,31] Better liver resection status [25]
Pulmonary resection		Well/moderate differentiation of tumor (histological grade) [32] Lower tumor marker CA19-9 levels pre- and/or post-treatment(s) [33,34]	Fewer and/or smaller lung lesions [34,35] Longer DFI [32,34,36]	
Ablation	Younger age [37] Performance status 0–1 [37]	Lower tumor stage [38] Tumor in pancreatic body/tail [39] Well/moderate differentiation of tumor (histological grade) [37,38,40] Neutrophil-to-lymphocyte ratio (NLR) < 2.5 [38]	Fewer and/or smaller liver/lung lesions [37,38,39,40,41] Longer DFI [38,42] Single-organ metastatic disease [38]	
Embolization	Male [43]	Smaller primary tumor size [44] Lower tumor marker CA19-9 levels pre- or post-treatment(s) [45] Absence of ascites [44] Decreased albumin and bilirubin prior to local treatment [46]	Fewer and/or smaller liver/lung lesions [47] Single-organ metastatic disease [48]	Primary tumor resection [46] Local metastatic treatment [44]

**Table 3:** Hepatic metastasectomy in mPDAC. NS = not specified; R = retrospective; Pro = prospective; PM = primary + metastatic resection; P = primary resection; M = metastatic resection; res = resection; chemo = chemotherapy; SE = surgical exploration; PB = palliative bypass; ILN = inter-aortocaval lymph node; LDT = liver-directed therapy; meta = metastasis; R0,1,2 = resection margin; Sc = synchronous; Mc = metachronous; N = neoadjuvant chemotherapy; A = adjuvant chemotherapy.

Authors	Year	Design	Nr. of Patients (Hepatic mPDAC)	Study Details	Resection Details	Synchronous/Metachronous	Morbidity, Grade 3+	Peri-Procedural Mortality	Chemotherapy, Neoadjuvant/Adjuvant	Median Overall Survival (Months), Hepatic mPDAC Only
Yamada [49]	2006	R	6	6 Primary + liver res (PM)	NS	5/1	NS	NS	NS	NS
Gleisner [50]	2007	R	68	17 Primary + liver res (PM) 66 Palliative bypass (control)	NS	88/0	NS	9%	N: NS A: 32%	From primary/meta res: -PM: 5.9 -Control (PB): 5.6
Shrikhande [31]	2007	R	10	29 Primary res ± ILN res or liver res or multi-organ res (PM)	R0: 86% R1: 14%	29/0	NS	0%	N: 3% A: 69%	From primary/meta res: -PM: 11.4 -Control (SE/PB): 5.9
De Jong [51]	2010	Pro	42	126 Primary res + LDT (incl. liver res, ablation, embolization) (PM)	NS	15/28	Sc: 12.4% Mc: 20.3%	Sc: 2% Mc: 3%	NS	From primary/meta diagnosis or resection (unknown): -PM: 13.6
Dünschede [52]	2010	Pro	23	13 Primary + liver res (PM) 10 Chemotherapy (control)	R0 pancreas: 100% R0 liver: Sc: 89% Mc: 100%	14/9	NS	Sc: 0% Mc: 0%	N: NS A: -PM: 50% -Control: 100%	Sc, from primary/meta diagnosis: -PM: 8 -Control (chemo): 11 Mc, from meta diagnosis: -PM: 31 -Control (chemo): 11
Klein [53]	2012	R	22	22 Primary + liver res (PM) 22 Primary res only (M0)	Liver: R0: 32% R1: 46% R2: 23%	22/0	18%	0%	N: NS A: 100%	From primary/meta diagnosis or resection (unknown): -PM: 7.5
Zanini [30]	2015	R	15	15 Primary + liver res (PM)	R0: 47% R1: 53%	11/4	Sc: 9% Mc: 25%	0%	N: NS A: 100%	From meta res: -Sc PM: 8.3 -Mc PM: 11.4
Bahra [54]	2015	R	29	21 Primary + liver res (PM) 24 Primary res only (control) 45 Chemotherapy (control)	R0M1: 27% R1M1: 27% R2M1: 13% R2M0: 33%	PM: 29/0 Control: NS	20%	2%	N: NS A: -PM: 98% -Control: 100%	From primary/meta res: -PM: 10.4 -Control (chemo): 7.2
Tachezy [24]	2016	R	138	69 Primary + liver res (PM) 69 Liver res only (control, M)	R0: 58% R1: 32% R2: 10%	138/0	PM: 7% SE: 5%	PM: 1% SE: 1%	N: -PM: 14% -Control: 1% A: -PM: 80% -Control: 82%	From primary/meta treatment: -PM: 14.5 -Control (M): 7.5
Hackert [55]	2016	Pro	85	85 Primary + liver res (PM) 43 Primary + ILN res	Liver: R0: 19% R1: 60% Rx: 21%	Liver: 62/23	NS	Sc: 3% Mc: 4%	N: 16% A: 75%	From meta res: -Sc + Mc PM: 12.3
Crippa [28]	2016	R	127	11 Primary ± liver res (PM) 116 Chemotherapy (control)	R0: 82% R1: 18%	3/8	NS	PM: 0% Control: NS	N: 100% A: -PM: 82%	From primary diagnosis: -Sc + Mc PM: 39 -Control (chemo): 11
Wright [56]	2016	R	16	23 Primary ± liver/lung res (PM)	R0: 91% R1: 9%	23/0	13%	0%	N: 100% A: NS	NS
Kim [29]	2016	R	45	35 Primary res ± metastasectomy (PM) 35 No res (matched controls)	NS	70/0	PM: 20% Control: 6%	0%	N: NS A: -PM: 83% -Control: 57%	NS
Andreou [25]	2018	R	76	76 Primary + liver res (PM)	R0: 82% R1: 18%	76/0	16%	5%	N: 5% A: 72%	NS
Kandel [57]	2018	R	18	6 M1PDAC: Primary res + meta res/RFA/embolization (PM) 18 M1PDAC: No res, chemo	M1R0: 83% M1R1: 17%	NS	NS	NS	N: -PM: 100% -Control: 44% A: 100% all groups	NS
Yang [26]	2020	R	89	48 Primary + liver res (PM) 10 Surgical exploration, chemo (control) 31 No res, chemo (control)	R0: 100%	89/0	NS	PM: 4%	N: 27% A: -PM: 79% -Control (SE): 100% -Control: 100%	From primary/meta res: -PM: 7.8 -Control (SE, chemo): 4.3 -Control (chemo): 7.6
Gu [27]	2020	R	73	36 Primary + meta res (PM) 60 Surgical exploration (control) 54 Palliative bypass (control)	R0: 94% R1: 6%	150/0	PM: 3% SE: 0% PB: 2%	PM: 0% SE: 3% PB: 4%	N: 0% A: -PM: 19% -Control (SE): NS -Control (PB): NS	NS
Schwarz [58]	2020	R	33	25 Primary + liver res (PM) 8 Primary res + chemo (matched controls)	Liver: R0: 96% R1: 4%	0/33	12%	0%	N: NS A: -PM: 88% -Control: 100%	From meta diagnosis: -PM: 36.8 -Controls (P + chemo): 9.2

**Table 4:** Pulmonary metastasectomy in mPDAC. NS = not specified; R = retrospective; PM = primary + metastatic resection; P = primary resection; M = metastatic resection; res = resection; CRT = chemoradiotherapy; chemo = chemotherapy; BSC = best supportive care; RFA = radiofrequency ablation; SBRT = stereotactic body radiotherapy; meta = metastasis; R0,1,2 = resection margin; LAPC = locally advanced pancreatic cancer; Sc = synchronous; Mc = metachronous; N = neoadjuvant chemotherapy; A = adjuvant chemotherapy. * From the pulmonary resection point of view. Neoadjuvant: after pancreatic resection or prior to pulmonary metastasectomy. Adjuvant: after pulmonary metastasectomy.

Authors	Year	Design	Nr. of Patients (Pulmonary mPDAC)	Study Details	Synchronous/Metachronous	Morbidity, Grade 3+	Peri-Procedural Mortality	Chemotherapy *, Neoadjuvant/Adjuvant	Median Overall Survival (Months), Pulmonary mPDAC Only
Arnaoutakis [59]	2011	R	31	9 Primary res + CRT + lung res (PM) 22 Primary res + CRT (control)	0/31	NS	Lung res: 0% Control: NS	N: 100% A: NS	From primary res: -PM: 51 -Control (P + chemo): 23
Thomas [36]	2012	R	7	14 Primary + meta res/RFA (7 lung, PM) 405 Primary res only (incl. lung, other sites)	0/7	NS	0%	N: 76% A: 29%	From primary res: -PM: 92.3
Downs-Canner [60]	2015	R	58	*Data available on 41 patients:* 8 Primary res/SBRT + lung res (PM) 23 Primary res + chemo (control) 10 Primary res + BSC (control)	0/58	NS	NS	N: 88% A: 50%	From primary diagnosis: -PM: 67.5 -Control (P + chemo): 33.8 -Control (P + BSC): 29.9 From meta diagnosis: -PM: 27 -Control (P + chemo): 18.9 -Control (P + BSC): 11.5
Robinson [33]	2016	R	16	15 Primary res + lung res (PM) 1 Primary SBRT + lung res (PM)	1/15	NS	0%	N: 88% A: 56%	From primary res: -PM: 52 From meta res: -PM: 28
Kruger [35]	2016	R	40	13 S Primary res + lung res (PM) 22 M Primary res + lung res (PM) 5 M CRT primary + lung res (LAPC, M)	13/27	NS	NS	N: 71% A: NS	From meta diagnosis: -Sc PM: 22.8 -Mc PM: 31.3 -Mc M: 10.7
Nakajima [61]	2017	R	16	16 Primary res + lung res (PM)	0/16	NS	0%	N: 59% A: 71%	From primary res: -PM: 92 From meta res: -PM: 37
Okui [62]	2017	R	6	6 Primary + lung res (PM)	0/6	NS	NS	N: 100% A: NS	*Median follow-up, since all patients were alive* From primary res: -PM: 81.7 From meta res: -PM: 37.3
Yasukawa [63]	2017	R	12	11 Primary res + lung res (PM) 1 CRT + lung res	0/12	NS	0%	N: 100% A: 100%	From primary res: -PM: 121 From meta res: -PM: 47
Ilmer [32]	2019	R	11	11 Primary + lung res (PM)	0/11	0%	0%	N: 91% A: 100%	From primary res: -PM: 37.7 From meta res: -PM: 26
Groot [34]	2019	R	96	19 Primary + lung res (PM) 77 Primary res only (controls): -45 CRT -32 BSC	0/96	Lung res: 0%	0%	N: -PM: 5% A: -PM: 53%	From primary res: -PM: 68.9 -Control (P + CRT): 34.2 -Control (P + BSC): 24.5 From meta res: -PM: 35 -Control (P + CRT): 20.2 -Control (P + BSC): 8.1
Kaiho [64]	2019	R	12	Primary + lung res (PM)	NS	NS	0%	N: -mPDAC: 75% A: -mPDAC: 75%	NS
Shimizu [65]	2020	R	13	6 Primary + lung res (PM) 7 Primary res only (control)	0/13	NS	0%	N: -PM: 100% -Control: 71% A: -PM: 50% -Control: 0%	From primary res: -PM: 39 -Control (P + BSC): 33

**Table 5:** Ablation in mPDAC. NS = not specified; R = retrospective; PM = primary + metastatic locoregional treatment; P = primary locoregional treatment; M = metastatic locoregional treatment; res = resection; chemo = chemotherapy; N = neoadjuvant; A = adjuvant; Ac = acute toxicity; L = late toxicity; Sc = synchronous; Mc = metachronous; meta = metastasis; RFA = radiofrequency ablation; IRE = irreversible electroporation; SBRT = stereotactic body radiotherapy; HIFU = high intensity focused ultrasound; LN = lymph nodes; LR = local recurrence; LAPC = locally advanced pancreatic cancer; tox = toxicity; CRT = chemoradiotherapy.

Authors	Year	Design	Nr. of Patients (mPDAC)	Study Details	Synchronous/Metachronous	Morbidity, Grade 3+	Peri-Procedural Mortality	Chemotherapy, Neoadjuvant/Adjuvant	Median Overall Survival (Months), mPDAC Only
***RFA***									
Park [40]	2012	R	34 liver	34 Primary res + liver RFA (PM)	6/28	NS	0%	N: 68% A: 62%	From primary res: -PM: 18 From meta RFA: -PM: 14
Hua [39]	2017	R	102 liver	102 no primary res (unresectable) + liver RFA (M)	102/0	0%	0%	N/A: 100% *Unclear whether prior to or after liver RFA*	From primary/meta diagnosis: -M: 11.4
Lee [38]	2020	R	126 liver	60 Primary res + liver RFA (PM) 66 Primary res + chemo for meta (control, P)	0/126	13%	0%	N: 80% A: NS	From meta RFA: -PM: 12 -Control (P + chemo): 9.1
***IRE***									
Hong [67]	2018	R	7 liver, peritoneum, omentum	Primary res + meta IRE (PM) *or* Primary IRE + metastasectomy (PM)	7/0	NS	0%	N: 100% A: 57%	From initial local treatment: -PM: 16
**Authors**	**Year**	**Design**	**Nr. of Patients (mPDAC)**	**Study Details**	**Synchronous/Metachronous**	**Morbidity,** **acute/late, Grade 3+**	**Peri-Procedural Mortality**	**Chemotherapy,** **Neoadjuvant/Adjuvant**	**Median Overall Survival (Months),** **mPDAC Only**
***SBRT***									
Chang [68]	2009	R	15 *Metastatic sites not specified*	15 primary SBRT only (P)	15/0	Ac: 1%	0%	Prior: 19% Concurrent: 77%	From primary SBRT: -P: 10.5
Su [69]	2015	R	16 *Metastatic sites not specified*	16 Primary SBRT only (P)	16/0	Ac: 0% L: NS	0%	N: 8% A: 8%	From primary SBRT: -P: 8.5
Gkika [70]	2017	R	14 liver, LN	-5 Primary res + primary SBRT (P) -9 Primary res + meta SBRT (PM) -2 Primary SBRT + meta SBRT (PM) -2 Primary SBRT (P)	Sc + Mc *Numbers not specified*	Ac: 6% L: 6%	0%	N: NS A: 78%	NS
Lischalk [41]	2018	R	20 *Metastatic sites not specified*	20 Primary SBRT only (P)	20/0	Ac: NS L: 0%	0%	N: 60% A: 100%	From primary SBRT: -P: 13.6
Scorsetti [42]	2020	R	41 liver, lung, LN	33 Primary res (± CRT) + meta SBRT (PM) 8 Meta SBRT only (M)	2/39	NS	NS	N: 83% A: 22%	From SBRT: -M ± P: 23
***HIFU***									
Li [37]	2016	R	120 liver, lung, LN	61 HIFU meta + chemo ± primary res (M ± P) 59 Chemo ± primary res (control, ± P)	NS	0%	0%	Concomitant: 100%	From meta HIFU/chemo: -M ± P: 10.3 -Control (chemo ± P): 6.6

**Table 6:** Embolization in mPDAC. NS = not specified; R = retrospective; Pro = prospective; TARE = transarterial radioembolization; SIRT = selective internal radiation therapy (same as TARE); TACE = transarterial chemoembolization; PM = primary + metastatic locoregional treatment; P = primary locoregional treatment; M = metastatic locoregional treatment; res = resection; RFA = radiofrequency ablation; ISI = iodine-125 seed implantation; LDT = liver-directed therapy; N = neoadjuvant; A = adjuvant; EHD = extrahepatic disease; Sc = synchronous; Mc = metachronous.

Authors	Year	Design	Nr. of Patients (mPDAC)	Study Details	Synchronous/Metachronous	Morbidity, Grade 3+	Peri-Procedural Mortality	Chemotherapy, Neoadjuvant/Adjuvant	Median Overall Survival (Months), mPDAC Only
***SIRT/TARE***									
Cao [71]	2010	Pro	7 liver (±EHD)	3 Primary res + liver SIRT (PM) 4 liver SIRT only (M)	6/1	Ac: 0% L: 0%	0%	N: 100% A: NS	NS
Michl [45]	2014	R	19 liver (±EHD)	15 Primary res + liver SIRT (PM) 4 liver SIRT only (M)	9/10	Ac: 9% L: 43–64%	16% (likely TARE related)	N: 84% A: 47%	From meta SIRT: -M ± P: 9
Gibbs [72]	2015	Pro	14 liver (±EHD)	4 Primary res + liver SIRT (PM) 10 liver SIRT only (M)	Sc + Mc *Numbers not specified*	Ac: 36% L: 50%	14%	Concomitant: 100%	From enrolment/SIRT: -PM: 13.6 -M: 4.2
Kim [73]	2016	R	16 liver (±EHD)	6 Primary res/SBRT + liver SIRT (PM) 10 liver SIRT only (M)	NS	6%	0%	Concomitant: 94%	From meta diagnosis: -M ± P: 22 From meta SIRT: -M ± P: 12.5
Kim [46]	2019	R	33 liver (±EHD)	23 Primary res/SBRT + liver SIRT (PM) 10 liver SIRT only (M)	NS	Clinical: 15% Lab: 9%	3% (likely TARE related)	N: 82% A: 30%	From primary diagnosis: -M ± P: 20.8 From meta SIRT: -M ± P: 8.1
Nezami [74]	2019	Pro	3 liver	3 Primary treatment NS + liver SIRT (M ± P)	NS	Clinical: 38.5% Lab: 38.5%	0%	Concomitant: 100%	NS
Kayaleh [47]	2020	R	26 liver (±EHD)	8 Primary res + liver SIRT (PM) 18 no primary res + liver SIRT (M)	13/13	Clinical: 3 in 77 pts Lab: 9 in 77 pts	0%	N: 100% A: 73%	From primary diagnosis: -M ± P: 33 From meta diagnosis: -M ± P: 21.8 From meta SIRT: -M ± P: 7
***TACE***									
Kim [75]	2010	R	15 liver	15 Primary res + liver TACE (PM)	0/15	13%	0%	N: 13% A: NS	From meta diagnosis: -PM: 9.6 From meta TACE: -PM: 7.5
Azizi [43]	2011	R	32 liver	32 Primary res + liver TACE (PM)	NS	0%	0%	N: 100% A: NS	From meta TACE: -PM: 16
Kotoyan [76]	2012	Pro	6 liver (± EHD)	6 Primary NS + liver TACE (M ± P)	NS	30%	0%	N: 100% A: NS Concomitant: 100%	From unknown: -M ± P: 9.3
Sun [48]	2017	R	18 liver (± EHD)	18 liver TACE ± primary res (M ± P)	NS	0%	0%	N: 44% A: NS	NS
Vogl [77]	2018	R	112 liver	112 Primary res + liver TACE (PM)	NS	0%	0%	N: 100% A: NS	From TACE: -PM: 19
Das [78]	2019	R	182 liver	-84 RFA/ISI + TACE -59 TACE -123 matched control: syst chemo *All groups include M1 and M0 disease*	NS	RFA/ISI + TACE: 13 in 75 pts TACE only: 16 in 143 pts Controls: 28 in 123 pts	0%	N: NS A: NS Control: 100%	NS
***TARE/TACE***									
Ouyang [44]	2018	R	184 liver (±EHD)	*No primary resection, some pts may have received primary SIRT* -64 LDT + systemic chemo (M): ○ 20× TARE ○ 14× TACE ○ 17× TARE + TACE ○ 13× other combinations -120 Systemic chemo only (control)	184/0	M: 30% Control (chemo): 18%	TACE: 1 pt	N: 100% A: NS	From primary/meta diagnosis: -M: 8.7 -Control (chemo): 6.3

**Table 7:** Survival outcomes per treatment group: no (CRT, BSC), single (P or M) or double (P + M) local treatments. mOS = median overall survival; P = local primary pancreatic treatment; M = local metastatic treatment; CRT = chemo(radio)therapy; BSC = best supportive care; Sc = synchronous; Mc = metachronous. * Data from Liu et al. [66], not included in the articles selected for this systematic review.

(Locoregional) Treatments	Hepatic mPDAC mOS from Metastatic Diagnosis/Treatment	Pulmonary mPDAC mOS from Metastatic Diagnosis/Treatment
P + M	7.8–19 (Sc + Mc)	22.8–47 (Sc + Mc)
P only	9.1–9.2 (Mc)	8.1–20.2 (Mc)
M only	7.5 (Sc)	10.7 (Mc)
CRT/BSC	4.3–7.6 (Sc + Mc)	11.8 * (Sc + Mc)

## Data Availability

The data presented in this study are available on request from the corresponding author.
